# Depression and Parkinson's disease: a Chicken-Egg story

**DOI:** 10.3934/Neuroscience.2022027

**Published:** 2022-11-23

**Authors:** Ashok Chakraborty, Anil Diwan

**Affiliations:** AllExcel, Inc. Shelton, CT, USA

**Keywords:** Parkinson's disease, depression, neuro degeneration, Aetiology, Dopamine

## Abstract

Parkinson's disease (PD) is a neurodegenerative disease, however, besides the motor symptoms, such as rest tremor, hypokinesia, postural instability and rigidity, PD patients have also non-motor symptoms, namely neuropsychiatric disorders. Apart from the required motor symptoms, psychopathological symptoms are very common and include mood disorders, anxiety disorders, hallucinations, psychosis, cognitive deterioration and dementia. The underlying pathophysiological process in PD is mainly due to the loss of dopaminergic neural cells and thereby causes the shortage of nigrostriatal dopamine content in them. In addition, it may involve other neurotransmitter systems such as the noradrenergic, serotonergic, cholinergic and noradrenergic systems as well. Depression can result from any unhealthy conditions making the diagnosis a challenging task. The manifestation of depression associated with or without PD is inadequate. The co-occurrence of depression and PD often leads to the conceptual discussion on whether depressive symptoms appear before or after PD develops. This paper will discuss the conceptual mechanism of PD and depression. Keep in mind both conditions belong to two separate entities but share some similar aspects in their pathophysiology.

## 1. Introduction

Parkinson's Disease (PD) is one of the major neurodegenerative diseases that commonly start during the middle age of life, even though early onset of the disease has happened [1]. Only 14–15% of cases of PD while found to be linked with genetic abnormalities, most of the PD cases are still known as sporadic, however, related to age, brain injury, pesticides, etc [2]. The major symptoms of PD are tremors, slow movement, muscle weakness and ultimately memory loss [3]. The pathology occurs due to the lack of dopamine (DA) in the substantia nigra (SN) region of the brain, as well as the presence of aggregated α-synuclein (Lewy bodies), neurofilaments and ubiquitin across the neural cell transmission pathways [7]–[9]. It has been calculated that in human when they lose 48–68% of the dopaminergic neurons at the SN and/or loss of DA content around 70–80% at the striatum occurs, PD may develop in them [10],[11].

Depression is a mental health disorder characterized by loss of interest in activities in daily life, and also loss of appetite, energy level, concentration, daily behavior, and sometimes thoughts doing suicide, also. Possible causes of PD are still unknown, but it is believed that some biological and/or social sources of distress may cause changes in certain neural circuits in the brain [4]. However, expression is not age-related and has a number of symptoms in common with PD [5]. These shared symptoms of PD and depression include tiredness, reduced energy, psychomotor retardation and a lack of facial expressions, loss of appetite, mental slowing, insomnia and difficulties in concentrating [6]. Further, lowered mood, anhedonia, and lack of interest, which can be found both in depression disorder and in PD [7]. The prevalence of depression in PD is 2 to 3 times higher than the major depressive disorder in the elderly population [8]–[11].

**Figure 1.**
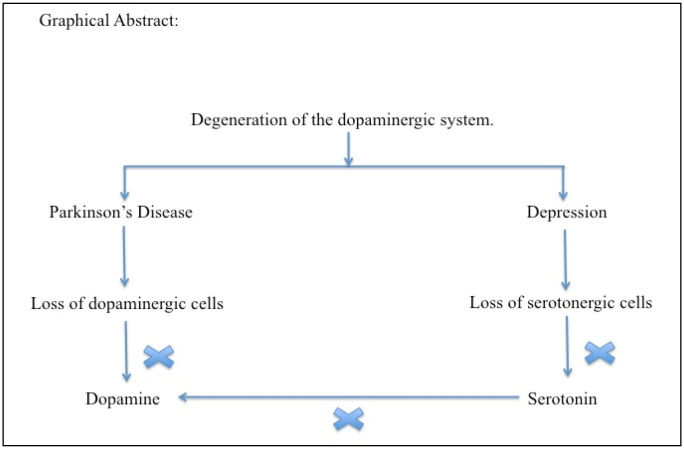
Graphical abstract.

### 1.1. Link between depression and Parkinson's Disease

- Depression and anxiety occur with high frequency in patients with Parkinson's disease. In fact, depression has been seen in nearly 50% of PD cases [8]–[11].
- Stress increases the death of dopaminergic cells and results in severe PD symptoms [12]–[15].
- Dopamine can help PD and major depression disorder [16].
- Loss of serotonin and dopamine can cause depression, a loss of energy and pleasure when doing any work. Furthermore, dopamine deficiency results in PD-specific symptoms, such as tremors, muscle weakness and difficulty with balance [12]–[20].
- Depression can result in memory loss or confusion, which also appears to PD patients later on [21].
- So far, depression has not been linked with any specific genotype of PD [22].

## 2. Etiology

Some studies have shown that PD constitutes to a biological risk factor for depression. Other studies conclude that depression also predisposes for PD [23],[24]. It is hypothesized that an allostatic state can developed due to depression, which leads to atrophy of nerve cells in the brain and cause neurodegenerative diseases [25]. There is also evidence that PD symptoms and its therapeutic regimen, a higher dose of levodopa, may increase the risk of depression in PD (reword sentence it doesn't make sense) There is evidence that a higher dose of levodopa may increase the risk of depression in PD [26]–[30].

The general consensus of the case histories on depression induced by dopa-agonists is to improve mood. Recent publications of the antidepressant effect of pramipexole in patients with PD have led to clinical investigations of the usefulness of pramipexole in the treatment of depression in patients without PD [31]–[33].

Several hypotheses try to provide a pathophysiological explanation for the higher prevalence of depression in PD patients. However, none have been empirically tested so far. Mayeux et al. formulated the serotonergic hypothesis in 1984 based on the findings that serotonergic activity in the cerebrospinal fluid and the brains of PD patients was lowered [34].

### 2.1. Biochemical Theory of Depression

The serotonergic and dopaminergic hypothesis indicates that serotonin inhibits dopamine release but eventually causes the onset of PD symptoms. At the same time, it is known that a reduced serotonergic tone is a risk factor for depression [12],[13]. Furthermore, the dopaminergic hypothesis considers depression is caused by the degeneration of the mesolimbic and mesocortical structures of the dopaminergic system. This may explain why depression may occur in patients with or without PD [14].

### 2.2. Genetics in PD and in Depression

**Table 1.** Genetic factors in PD and Depression.

Gene(s)	PD	Depression
*PARK2* Gene	Mutations in the *parkin* gene (PARK2) on chromosome 6q have been implicated in early onset PD (EOPD), commonly defined as PD with onset <50 years of age, but their role in non-motor manifestations is not well established [35]	Genotype was not associated with depression risk among probands. However, *parkin* mutations might be predisposed to depression prior to the onset of PD [38]
*LRRK2* Gene	A meta-analysis of studies investigating *LRRK2* rs34637584 confirmed that the minor allele carriers had significantly less cognitive impairment (*p* = 0.015) in people with PD [36]	Minor alleles of *GBA* variants rs76763715, rs421016, rs387906315 and rs80356773 were associated with more depressive symptoms in PD.
*APOE*, *BDNF, CRY1*	*APOE* ε4 allele has been associated with more cognitive impairment in PD [37]	*BDNF* (rs6265) and *CRY1* (rs2287161) variants have been associated with more depressive symptoms in people with PD. [38]

Recently, a genetic abnormality behind the PD was reviewed by Chakraborty and Diwan [39]. A genome-wide association study (GWAS) with million people have identified 178 gene variants linked to major depression [40]–[42]. These types of large-scale findings help the clinicians:

- to evaluate the polygenic risk scores, and
- to develop new medications.

### 2.3. Environmental factors in PD and Depression

It was suggested that PD is linked to numerous environmental toxins for example, chemicals [43]–[45], pesticides [46] and heavy metals [47]–[49]. Ambient air pollution from traffic can also augment the chance of PD onset [50],[51]. Long-time use of illicit drugs can cause abnormal morphology in the *substantia nigra* [52], and produces reactive oxygen species causing dopamine neuron toxicity and death [53],[54].

**Psychological factors**

**Negative thoughts**, like sadness, helplessness and hopelessness perceived for long periods may make a person more vulnerable to depression [55].

**Social isolation**: Social Isolation or a lack of a supportive social network, early retirement or loss of independence can increase the depression risk, too [56].

### 2.4. The Link Between Depression and PD

Depression is a part of Parkinson's disease itself. PD affects the areas of the brain that produce dopamine, norepinephrine and serotonin—chemicals involved in regulating energy, mood, mood, energy, motivation, sleep and appetite [57]. For many people, the challenges of Parkinson's disease are enough to cause depression [57].

On the other hand, the pathological process of Parkinson's disease and the mood disorder, like depression and bipolar disorders, both results from the same brain cell damage beneath the substantia niagra. These cells could be affected years before the tremors are even evident. This finding means that depression may precede a formal diagnosis of PD.

### 2.5. Clinical Features of PD and Depression (Table 2)

**Table 2.** Clinical features of PD and Depression.

	Major depression	Parkinson's disease
Motor phenomena	Psychomotor retardation, Stooped posture, Restricted/depressed affect, Agitation	Bradykinesia, Stooped posture, Masked face Hypomimia, Tremor
Other somatic complaints	Physical complaints, Muscle tension, Gastrointestinal symptoms, Sexual dysfunction	
Vegetative changes	Decreased energy, Fatigue, Sleep and Appetite changes	
Cognitive disturbances	Poor concentration, Decreased memory, Impaired problem-solving	

### 2.6. Other Commonness in Depression and PD:

- Long-time psychiatric disorders, like depression, anxiety may end up in motor nerve illness resulting movement disorder [58].
- PD victims experience depression and/or anxiety two to five years before a Parkinson's diagnosis, indicating depression could be a part of the underlying disease process [59].
- PD and depression affect the same part of the brains which is involved in thinking and emotion (SN region). Damage of SN region impacts the levels of three important neurotransmitters (dopamine, serotonin and norepinephrine) that influence mood and movement [60].
- In one study [61] of late onset PD, 9.2% of patients had a history of depression at the time of a diagnosis of PD, much higher than the control cases (only 4%).
- Degeneration of dopaminergic fibers has been suggested to be involved in depression [62].
- There is evidence that noradrenaline may be involved in depression [63].

## 3. Treatment

Treatment of depression in PD patients is just as important as the treatment of PD itself because depression negatively affects cognitive performance, daily activities and quality of life [17],[18]. The treatment for mild depression is supportive psychotherapy, which may stimulate the patient to engage in personal and social activities. In cases of more severe depression, pharmacological treatment is warranted [19]. Dopamine reuptake inhibitor is the treatment of choice when it comes to depression in PD patients [19].

In the dopaminergic theory, depression is believed to be a result of the deficient self-reward mechanisms that are located in the mesocortical and mesolimbic dopaminergic structures [20]. Parkinsonism may occur occasionally during the treatment of depression in a patient who does not suffer from PD symptoms [21].

**Table 3.** Treatment strategies of PD and Depression [64]–[68].

Medication	Depression	PD
Dopamine supplementation	Helps depression	Helps PD
Selective serotonin reuptake inhibitors (SSRIs) Serotonin-norepinephrine reuptake inhibitors (SNRIs)	Several non-SSRI antidepressants used to treat depression (Effexor, Remeron. Webutrin, Amoxapine)	Most common type prescribed to people with Parkinson's disease.
Psychological Therapy (Cognitive-behavioral therapy, CBT)	CBT has been shown to reduce symptoms of depression by helping people change negative thinking patterns and behaviors.	Also helps PD symptoms
Regular exercise and healthy lifestyle	Helps lessen feelings of depression as well as PD symptoms	
A well-balanced diet Limited alcohol drinking No smoking	Helps with the feelings of depression and PD symptoms	
Transcranial Magnetic Stimulation (TMS)	An FDA-approved treatment for depression	
Non-Conventional and Complementary Therapies	**Complementary therapies** are designed to support traditional treatments	

## 4. Discussion and conclusion

- Parkinson's disease is a neurological disorder that involves an imbalance when standing and walking, tremors, stiff muscles and slow movement.
- Depression is a medical problem which can cause a long-lasting feelings of sadness or hopelessness.
- Many people experience sadness or grief when they receive a diagnosis of a serious condition such as Parkinson's disease. In some cases, depression can occur.
- Depression is a mood disorder that can affect a person's ability to carry out daily activities. About 50% of people with PD have depression at some time during their illness, and around 40% experience anxiety. This appears to be distinct from feeling sad knowing the diagnosis and prognosis of the disease.
- Reduced dopamine leads to the physical symptoms of Parkinson's disease [17],[30].
- Distinct diagnosis of PD and depression is challenging. Common symptoms of PD and depression include drooping eyes, having a flat expression, signs of apathy and slow speech that can occur.
- The levels of cerebrospinal fluid 5-HIAA, which is a metabolite of serotonin (5-HT), are reduced in depressed patients with or without PD symptoms [69],[70].
- Serotonin re-uptake inhibitors have no beneficiary effect on motor functioning but can be effective in treating depression [71]–[75].
- Tricyclic antidepressants (TCAs) can be used effectively for treating a motor disability in PD [71]–[73].
- Due to the overlapping symptomatology of PD and depression, it is often difficult to recognize depression as a separate entity. In fact, the partly shared pathophysiology may increase the difficulty when specifically treating mood symptoms, without influencing motor or cognitive symptoms.
- Large placebo-controlled studies are necessary to further evaluate the potential efficacy of the antidepressant treatment and allow evidence-based treatment guidelines to develop.
- Defects in the serotonergic neurotransmitters circuit can occur even without the involvement of dopaminergic neurons.
- The pathophysiology of “depression” in patients with or without PD should be better investigated.
- Altogether, it is common for a person with PD to experience symptoms of depression. It may stem from some of the same brain changes that cause the physical characteristics of PD. A doctor can help the individual in finding treatments for managing depression.
